# Exploring how genetic variation within Aquaporin‐4, influences the relationship between sleep and amyloid beta

**DOI:** 10.1002/alz.092408

**Published:** 2025-01-03

**Authors:** Tenielle Porter, Ayeisha Milligan Armstrong, Vincent Dore, Eleanor K. O’Brien, Pierrick Bourgeat, Paul Maruff, Christopher C. Rowe, Stephanie Rainey Smith, Victor L Villemagne, Simon M. Laws, The AIBL Research Group

**Affiliations:** ^1^ Centre for Precision Health, Edith Cowan University, Joondalup, Western Australia Australia; ^2^ Curtin Medical School, Curtin University, Bentley, Western Australia Australia; ^3^ Collaborative Genomics and Translation Group, School of Medical and Health Sciences, Edith Cowan University, Joondalup, Western Australia Australia; ^4^ Australian E‐Health Research Centre, CSIRO, Melbourne, VIC Australia; ^5^ Department of Molecular Imaging, Austin Health, Melbourne, VIC Australia; ^6^ The Australian e‐Health Research Centre, Commonwealth Scientific and Industrial Research Organisation, Brisbane, QLD Australia; ^7^ Cogstate Ltd., Melbourne, VIC Australia; ^8^ Florey Institute of Neuroscience and Mental Health, Parkville, VIC Australia; ^9^ Molecular Research and Therapy, Austin Health and University of Melbourne, Heidelberg, VIC Australia; ^10^ Florey Department of Neuroscience and Mental Health, University of Melbourne, Parkville, VIC Australia; ^11^ Murdoch University, Murdoch, Western Australia Australia; ^12^ The Florey Institute of Neuroscience and Mental Health, Melbourne Australia; ^13^ University of Pittsburgh, Pittsburgh, PA USA

## Abstract

**Background:**

The glymphatic system has been suggested as an important clearance mechanism for amyloid‐β (Aβ) during sleep. Animal and cellular models have suggested this clearance mechanism involves the water‐channel protein, Aquaporin‐4 (encoded by the *AQP4* gene), located primarily in the astrocytic end‐feet. We have previously reported on the interaction between genetic variants within *AQP4*, sleep and cross‐sectional cortical amyloid‐β (Aβ) burden. This study investigated how the relationship between *AQP4* genetic variants and sleep may further influence changes in cortical Aβ levels.

**Methods:**

The current study assessed cognitively unimpaired individuals enrolled in the Australian Imaging, Biomarker and Lifestyle (AIBL) Study of Ageing, who were considered Aβ accumulators (n = 319). Aβ accumulators were defined as those who had a high baseline level of cortical Aβ (centiloid ³20), or a positive rate of change in cortical Aβ, calculated using a minimum of 3 study time‐points. 13 *AQP4* gene variants were selected for analysis based on fine mapping of the gene region, and sleep was assessed using measures calculated from the Pittsburgh Sleep Quality Index (PSQI) questionnaire. The relationship between *AQP4* gene variants and rate of Aβ accumulation was investigated using linear regressions. Additional linear regression models with *AQP4* variant x sleep measure interaction terms and post‐hoc simple slopes were analysed to assess how these relationships may influence Aβ accumulation.

**Results:**

*AQP4*‐rs68006382 was associated with Aβ accumulation, where minor allele carriers/homozygotes were observed to have a higher rate of accumulation when compared to major allele homozygotes. Linear regressions with *AQP4* variant x sleep measure interaction terms revealed that in *AQP4*‐rs68006382 minor allele carriers there was a significant relationship between Aβ accumulation and sleep quality. Additionally, interaction analysis revealed a relationship between Aβ accumulation and sleep latency in *AQP4*‐rs3875089 minor allele carriers.

**Conclusion:**

The results of this study confirm our previous findings and suggests that interactions between *AQP4* genotypes and sleep measures are also associated with the rate of Aβ accumulation.

